# Increased activity of MAP, p70S6 and p90rs kinases is associated with AP-1 activation in spontaneous liver tumours, but not in adjacent tissue in mice

**DOI:** 10.1054/bjoc.1999.1040

**Published:** 2000-02-01

**Authors:** J Ostrowski, M Woszczynski, P Kowalczyk, T Wocial, E Hennig, L Trzeciak, P Janik, K Bomsztyk

**Affiliations:** Department of Gastroenterology, Medical Center of Postgraduate Education in the Maria Sklodowska-Curie Memorial Cancer Center and Institute of Oncology, 02–781 Warszawa, Roentgena 5, Poland; Departments of Animal Genetics and Cell Biology, Cancer Center and Institute of Oncology, Warsaw, Poland; Department of Medicine, University of Washington, Seattle, WA, USA

**Keywords:** MAPK, p706Sk, p90rsk, AP-1, ODC, EGF, liver neoplasms

## Abstract

Growth factor-responsive protein kinases regulate expression of genes involved in cell cycle control, cell proliferation and differentiation. To better understand the role of these kinases in the abnormal proliferation of malignant cells, we examined basal and epidermal growth factor (EGF)-inducible mitogen-activated protein kinase (MAPK), p70S6k and p90rsk activities in spontaneous hepatocellular neoplasms (adenomas and carcinomas) from CBA-T6 mice and in L1 sarcoma tumours implanted in livers of BALB/c mice. In spontaneous and implanted hepatic tumours, basal cytoplasmic and nuclear MAPK, p70S6k and p90rsk activities were significantly higher compared to the activities found in the part of the liver uninvolved by the tumour. Interestingly, the activities of these enzymes in the uninvolved tissue of the livers harbouring the tumour were higher compared to the livers from control mice. Basal kinase activities correlated with tumour morphology; they were lower in adenomas than in carcinomas and sarcomas. In contrast to basal activities, EGF-triggered kinase responses in normal livers and hepatic tumours were indistinguishable. Activating protein-1 (AP-1) DNA-binding activity was detected in tumours but not in the adjacent tissues. Constitutively activated kinases and AP-1 transcription factor found in hepatic malignancies are reminiscent of cells activated by EGF, suggesting that EGF and its intracellular effectors play a role in these malignancies. © 2000 Cancer Research Campaign


British Journal of Cancer (2000) 82(5), 1041–1050
© 2000 Cancer Research Campaign
DOI: 10.1054/ bjoc.1999.1040, available online at http://www.idealibrary.com on IDEAL®

### Increased activity of MAP, p70S6 and p90rs kinases is associated with AP-1 activation in spontaneous liver tumours, but not in adjacent tissue in mice

**J Ostrowski[1], M Woszczynski[2], P Kowalczyk[1], T Wocial[1], E Hennig[1], L Trzeciak[1], P Janik[3] and K Bomsztyk[4]**

[1]Department of Gastroenterology, Medical Center of Postgraduate Education in the Maria Sklodowska-Curie Memorial Cancer Center and Institute of Oncology, 02–781 Warszawa, Roentgena 5, Poland; Departments of [2]Animal Genetics and [3]Cell Biology, Cancer Center and Institute of Oncology, Warsaw, Poland; [4]Department of Medicine, University of Washington, Seattle, WA, USA

**Summary** Growth factor-responsive protein kinases regulate expression of genes involved in cell cycle control, cell proliferation and differentiation. To better understand the role of these kinases in the abnormal proliferation of malignant cells, we examined basal and epidermal growth factor (EGF)-inducible mitogen-activated protein kinase (MAPK), p70S6k and p90rsk activities in spontaneous hepatocellular neoplasms (adenomas and carcinomas) from CBA-T6 mice and in L1 sarcoma tumours implanted in livers of BALB/c mice. In spontaneous and implanted hepatic tumours, basal cytoplasmic and nuclear MAPK, p70S6k and p90rsk activities were significantly higher compared to the activities found in the part of the liver uninvolved by the tumour. Interestingly, the activities of these enzymes in the uninvolved tissue of the livers harbouring the tumour were higher compared to the livers from control mice. Basal kinase activities correlated with tumour morphology; they were lower in adenomas than in carcinomas and sarcomas. In contrast to basal activities, EGF-triggered kinase responses in normal livers and hepatic tumours were indistinguishable. Activating protein-1 (AP-1) DNA-binding activity was detected in tumours but not in the adjacent tissues. Constitutively activated kinases and AP-1 transcription factor found in hepatic malignancies are reminiscent of cells activated by EGF, suggesting that EGF and its intracellular effectors play a role in these malignancies. © 2000 Cancer Research Campaign

**Keywords:** MAPK; p706Sk; p90rsk; AP-1; ODC; EGF; liver neoplasms

The emergence of a neoplasm results from accumulated mutations of genes that encode factors involved in signal transduction, cell proliferation and other cellular processes. Protein kinases are key intracellular transducers of mitogenic signals that trigger gene expression and cellular proliferation (Carpenter et al, 1990). It is thought that a variety of proliferative disorders, such as cancer, might reflect abnormalities in signal transduction (Levitzki, 1996).

ERK is a component of one of the mammalian mitogen-activated protein kinase (MAPK) cascades (reviewed in Lewis et al, 1998). The MAPK pathway is triggered by a surface receptor through the action of the guanosine 5′-triphosphate(GTP)-binding protein p21 Ras. Activated Ras binds and activates the c-Raf protein kinase that, in turn, phosphorylates and activates MAPK kinase (MEK). MEK then phosphorylates and activates MAPK. The rapid activation of the MAPK cascade and the phosphorylation of its targets plays a pivotal role in the signal transduction pathway activating quiescent cells to re-enter the cell cycle at G1.

The phosphorylation of the 40S ribosomal proteins S6 is another early event associated with mitogenic response (Thomas et al, 1979; Decker, 1981). Its phosphorylation is mediated by 70 kDa and 90 kDa ribosomal S6 kinases denoted p70S6k and p90rsk respectively (Banerjee et al, 1990; Kozma et al, 1990; Moller et al, 1994). Both p70S6k and p90rsk are active in vivo towards specific five-serine residue of the S6 proteins (Pullen and Thomas, 1997). While p70S6k is relatively specific for phosphorylation of ribosomal S6 protein, the p90rsk has a wider range of substrates including proteins that contain consensus phosphorylation site, R-X-X-S (Sturgil and Wu, 1991). Although both enzymes share amino acids sequence similarity, they are regulated by different kinase cascades. Whereas p90rsk is activated by the Ras-dependent MAPK pathway (Marshall, 1994), activation of p70S6k requires serial multisite phosphorylations by proline-directed kinases (such as MAPK), an unidentified PtdIns(3,4,5)-P3-dependent kinase, and constitutively active PDK1 (Downward, 1998).

Stimulation of cells with epidermal growth factor (EGF) can result in cellular proliferation, morphogenesis and chemotaxis. EGF is a potent mitogen for hepatocytes in vitro (McGowan et al, 1981). It is also produced by the liver during the immediate–early phase of the liver growth (Mullhaupt et al, 1994) and functions in mice as a mitogen during liver regeneration (Fausto et al, 1995). Recently, we have shown that cytoplasmic and nuclear MAP, p70S6 and p90rs kinases are activated after intraperitoneal injection of EGF in mouse liver (Ostrowski et al, 2000) that may reflect a role of kinase cascades in liver regeneration and repair. Activator protein 1 (AP-1) transcription factor, a dimeric complex composed of the *Fos* and *Jun* families, regulates various aspects of growth and differentiation (Lee et al, 1987; Nadori et al, 1997). AP-1 DNA-binding activity was also stimulated by EGF.

ODC activity is a key regulatory enzyme in growth processes (Pegg, 1986). Sustained high level of ODC activity is thought to play a role in tumorigenesis. For example, increased expression of

*Received 16 June 1999*
*Revised 18 October 1999*
*Accepted 2 November 1999*

*Correspondence to:* J Ostrowski

ODC mRNA and high activity of ODC were observed in rat hepatocarcinogenesis (Huber et al, 1989) and in human hepatocellular carcinoma (Tamori et al, 1994). ODC activity was also correlated with growth rate in a panel of hepatoma cell lines (Williams-Ashman et al, 1972).

To gain more insight into the role of MAP, p70S6 and p90rs kinases, AP-1 and ODC in malignancies we examined the activity of these factors in spontaneous hepatocellular neoplasms in CBA-T6 mice and in liver-implanted L1 sarcoma tumours in BALB/c mice. We found that cytosolic and nuclear activities of these enzymes were higher in the tumours compared to the surrounding normal tissue. Surprisingly, the kinase activities in normal tissue of livers harbouring tumours were higher compared to the livers from normal control mice without a neoplasm. ODC activity closely followed the same pattern. Systemic administration of EGF into mice stimulated activity of these enzymes in normal liver tissue and tumours to the same level. AP-1 DNA-binding activity was increased in nuclear extracts from the neoplasms but was not detected in the adjacent hepatic tissue.

### MATERIALS AND METHODS

### Animals and treatment protocol

Mice housed at Cancer Center, Warsaw, breeding facilities were maintained under constant room temperature with a 12–12-h light–dark cycle and permitted free access to water and to standard food pellets. The animals received humane care in compliance with the regulations of Cancer Center. We used CBA-T6/W mice which had developed spontaneous hepatocellular neoplasms. Control CBA-T6/W mice were 24 months of age.

Experiments also utilized BALB/c mice with liver-implanted L1 sarcoma tumours. L1 sarcoma cell line was propagated under standard conditions in modified Eagle's medium (MEM) supplemented with 10% fetal calf serum (FCS) and antibiotics (Janik et al, 1981). Two-month-old BALB/c mice were anaesthetized with ether for laparotomy and $10^5$ viable L1 cells, suspended in 0.05 ml of phosphate-buffered saline (PBS) were injected under the capsule of the left lateral lobe of the liver. There was no perioperative mortality. Three weeks later, animals with implanted hepatic tumours and those sham-operated were used in these studies.

Ten minutes after intraperitoneal injection (10 μl $g^{-1}$ of body weight) of 0.9% saline or 0.9% saline containing EGF (1 mg $ml^{-1}$), mice anaesthetized with ether were sacrificed, the livers were rapidly resected and classified by gross examination into tumour and hepatic tissue. Two portions of each specimen were frozen in liquid nitrogen and stored in – 80°C until use. The remaining portions were fixed in formalin and embedded in paraffin for histological examination.

Cytoplasmic and nuclear extracts were prepared by a modified method of Dignam et al (1983) as described previously (Ostrowski et al, 1991) using frozen tissues which were pulverized under liquid nitrogen with a Mikro-Dismembrator II (B.Braun). In addition to dithiothreitol (DTT) (0.5 mM), phenylmethylsulphonyl fluoride (PMSF) (0.5 mM) and leupeptin (10 μg $ml^{-1}$), the lysis, extraction and dilution buffers contained the following phosphatase inhibitors: 30 mM p-nitrophenyl phosphate (pNPP), 10 mM sodium fluoride (NaF), 0.1 mM sodium orthovanadate ($Na_3VO_4$), 0.1 mM sodium molibdate ($Na_2MoO_4$) and 10 mM β-glycerophosphate.

Separation of the nuclear fraction from the cytosol was monitored with the cytosolic enzyme marker lactic dehydrogenase and the purity of nuclear extracts was greater than 92%.

The protein concentration was measured using MicroBCA protein assay (Pierce Chemical).

### Assay for p42–p44 MAPK, p706Sk and p90rsk activities

Immunocomplex kinase assays were performed as described previously (Ostrowski et al, 1998). Cytoplasmic and nuclear extracts containing 300 μg of total protein were immunoprecipitated with 5 μg of either anti-ERK1/anti-ERK2, anti-p70S6, or anti-p90rsk polyclonal antibodies (Santa Cruz Biotechnology) in immunoprecipitation buffer (150 mM sodium chloride (NaCl), 50 mM Tris–HCl, pH 7.5, 5 mM EGTA, 1% Triton X-100, 0.5% NP-40, 25 mM pNPP, 5 mM benzamidine, 1 mM PMSF, 20 mM NaF, 0.2 mM $Na_3VO_4$, 10 μg $ml^{-1}$ leupeptin) for 2 h at 4°C. Then 25 μl of protein A/G beads, which had been preincubated with immunoprecipitation buffer, were added and the samples were incubated overnight at 4°C on the rotator.

For MAPK immunocomplex assays, the beads were washed twice with immunoprecipitation buffer and twice with MAPK assay buffer (25 mM HEPES–NaOH (sodium hydroxide), pH 7.5, 10 mM β-glycerophosphate, 20 mM magnesium chloride ($MgCl_2$), 2 mM manganese chloride ($MnCl_2$), 0.1% Triton X-100, 0.1 mM DTT, 0.1 mM $Na_3VO_4$). Next, beads were mixed with 40 μl of MAPK assay buffer containing 20 μM ATP – 0.3 μCi [$\gamma^{32}$P]ATP, 2 μg protein kinase A (PKA) inhibitor, 2 μg PKC inhibitor, and 15 μg MBP peptide (APRTPGGRR). Phosphorylation reactions were carried out for 10 min at 30°C.

For p70S6k and p90rsk immunocomplex assays, the beads were washed twice with immunoprecipitation buffer and twice with p70/p90 assay buffer (25 mM Tris–HCl, pH 7.5, 10 mM β-glycerophosphate, 10 mM $MgCl_2$, 0.1 mM DTT, 0.1 mM $Na_3VO_4$). Next, beads were mixed with 40 μl of p70/p90 kinase assay buffer containing 20 μM ATP – 0.3 μCi [$\gamma^{32}$P]ATP, 2 μg PKA inhibitor, 2 μg PKC inhibitor and 15 μg S6 peptide (RRRLSSLRA). Phosphorylation reactions were carried out for 15 min at 30°C.

The reactions were terminated by adding 10 μl of 20% trichloroacetic acid (TCA). After centrifugation, the supernatants were spotted onto Whatman P-81 papers. The papers were washed several times in 0.5% phosphoric acid, rinsed with acetone, dried and counted for the radioactivity. Reaction blanks were prepared by using reaction mixture without peptide substrate. All assays were done in triplicates. The results were expressed in c.p.m. as radioactivity incorporated into MBP or S6 peptide.

### Assay of ornithine decarboxylase activity

ODC activity was assayed using a method previously described (Ostrowski et al, 1993). Frozen specimens were homogenized using Polytron PT1200 (Kinematica AG) in 300 μl of buffer containing 50 mM HEPES–NaOH (pH = 7.5), 2.5 mM DTT, 0.25 mM pyridoxal 5-phosphate and 0.1 mM EDTA. The reaction mixture consisted of 20 μl of homogenate's supernatant, 0.25 mM pyridoxal 5-phosphate, 2.5 mM DTT, 50 mM HEPES–NaOH (pH = 7.5), 0.1 mM EDTA with 0.2 μCi of L-[$^{14}$C]-ornithine hydrochloride (Amersham International) in a total volume of 40 μl. The reaction tube was sealed with plastic pipet tip containing a 0.5 cm × 4.0 cm piece of Whatman No. 1 filter paper soaked with 40 μl of β-phenylethylamine. Tubes were incubated

 *© 2000 Cancer Research Campaign*

at 37°C for 60 min and the reaction was then stopped by adding 200 µl of 2 M citric acid. After a further 60 min of incubation, the filter paper was removed, placed in 10 ml of scintillation liquid and counted in a scintillation counter. Results were expressed as pmol of $^{14}CO_2$ released per h mg$^{-1}$ of protein. All assays were performed in triplicate.

### Electrophoresis and immunoblotting

Equal amounts of sample protein (100 µg) were boiled in 2 × loading buffer (125 mM Tris–HCl, pH 6.8, 4% sodium dodecyl sulphate (SDS), 20% glycerol and 10% β-mercaptoethanol) (1:1, vol/vol) for 5 min. Proteins were separated by 10% or 12% SDS polyacrylamide gel electrophoresis (SDS-PAGE). After bathing the gel in transfer buffer (192 mM glycine, 25 mM Tris, 20% methanol, 0.005% SDS) for 30 min, proteins were electroblotted to polyvinyl difluoride (PVDF) membrane in transfer buffer. The membrane was blocked with 5% non-fat dry milk in TBST buffer (Tris-buffered saline containing 0.05% Tween-20) for 1 h at room temperature. Then, the membranes were probed for 1 h at room temperature with polyclonal anti-ERK-1/ERK-2, anti-p70S6k, anti-p90rsk, anti-EGF-receptor, anti-c-Jun, anti-JunB, anti-JunD, anti-c-Fos, anti-FosB, anti-Fra1, or anti-Fra2 antibodies (Santa-Cruz Biotechnology) diluted in 5% non-fat dry milk/TBST buffer. After washing, the membranes were incubated for 60 min at room temperature with secondary antibodies conjugated with alkaline phosphatase or horseradish peroxidase, and immunoreactions were detected using BCIP/NBT phosphatase substrate or enhanced chemiluminescence (ECL) Western blotting detection system.

### EGF receptor tyrosine phosphorylation

Cytoplasmic extracts containing 1 mg of total protein were immunoprecipitated with 10 µg of EGF receptor (EGFR) polyclonal antibodies (Santa-Cruz Biotechnology) in immunoprecipitation buffer for 2 h at 4°C. Then 25 µl of protein A/G beads, which had been preincubated with immunoprecipitation buffer, were added and the samples were incubated for 2 h at 4°C on the rotator. After centrifugation beads were washed four times with 1 ml of immunoprecipitation buffer and proteins were eluted by boiling in loading buffer. Eluted proteins were resolved on SDS-PAGE, and after electrotransfer, Western blotting was carried out with either an anti-phosphotyrosine or anti-EGFR antibodies.

### Electrophoretic mobility shift assay

The double-stranded oligonucleotide probe (sense strand, 5′-CTAGTGATGAGTCAGCCGGATC-3′) was end-labelled using [$\gamma^{32}$P]ATP and T4 polynucleotide kinase. Twenty micrograms of nuclear proteins were incubated in binding buffer (20 mM HEPES–NaOH, pH 7.9, 20% glycerol, 100 mM potassium chloride (KCl), 0.5 mM EDTA, 0.25% NP-40, 2 mM DTT, 0.1% PMSF) containing 2 µg poly(dI-dC), and $^{32}$P-labelled probe for 30 min at room temperature. The reactions were loaded onto a 6% native polyacrylamide gel. The gels were dried and autoradiographed.

### Statistical analysis

Results are presented as means ± standard deviation (s.d.). Significant difference between mean values was assessed by means of analysis of variance (ANOVA). *P*-values for differences from control results were calculated using NIR test. Means were considered to be different if $P < 0.05$.

### RESULTS

Spontaneous hepatocellular neoplasms are among the most common murine tumours. The intracellular processes responsible for these malignancies are poorly defined. To better understand these mechanisms we examined the activities of several kinases and other factors in 2-year-old CBA-T6 mice with spontaneous liver tumours histologically classified (Frith et al, 1994) as hepatocellular adenoma and hepatocellular carcinoma. We also studied 2-month-old BALB/c mice with transplantable L1 sarcoma implanted into the liver.

### MAPK activities in hepatic tumours, livers harbouring tumours and livers from control mice after intraperitoneal injection of either saline or EGF

Animals were treated with intraperitoneal injection of either saline or EGF. After ether anaesthesia livers were harvested, the tissues were histologically typed and frozen. Cytoplasmic and nuclear extract from frozen tissue were prepared and MAPK activities were measured by immunocomplex kinase assay. Results from these series of experiments are shown in Figure 1. In saline-treated mice (basal) both nuclear and cytoplasmic MAPK activities in liver adenomas and carcinomas were higher than the activities in the uninvolved part of the livers that harboured these tumours (cytoplasmic extracts (CE): adenoma vs adjacent liver $P < 0.01$; carcinoma vs adjacent liver $P < 0.05$), (nuclear extracts (NE): adenoma vs adjacent liver $P < 0.01$; carcinoma vs adjacent liver $P < 0.05$) or the livers from control animals (CE: adenoma vs control liver $P < 0.01$; carcinoma vs control liver $P < 0.05$), (NE: adenoma vs control liver $P < 0.01$; carcinoma vs control liver $P < 0.05$). Unexpectedly, these results also revealed that the MAPK activities are not only increased in the liver tumours but also in the surrounding histologically normal hepatic tissue (CE: liver harbouring adenoma vs control liver $P < 0.05$; liver harbouring carcinoma vs control liver $P < 0.01$), (NE: liver harbouring adenoma vs control liver $P < 0.01$; liver harbouring carcinoma vs control liver $P < 0.01$). Intraperitoneal injection of EGF not only increased cytoplasmic and nuclear MAPK activities in the livers of control animals and in the normal part of the livers of animals with the tumours but also in the tumours themselves (Figure 1). In fact, while there were demonstrable differences in the basal (saline-treated animals) MAPK activities between tumours and the normal tissues, the differences in the MAPK activities between tumours and the adjacent hepatic tissues from EGF-treated animals were not significant (CE: adenoma vs adjacent liver *P* = n.s.; carcinoma vs adjacent liver $P < 0.05$), (NE: adenoma vs adjacent liver *P* = n.s.; carcinoma vs adjacent liver *P* = n.s.), (CE: adenoma vs control liver *P* = n.s.; carcinoma vs control liver $P < 0.05$), (NE: adenoma vs control liver *P* = n.s.; carcinoma vs control liver *P* = n.s.), (CE: liver harbouring adenoma vs control liver *P* = n.s.; liver harbouring carcinoma vs control liver *P* = n.s.), (NE: liver harbouring adenoma vs control liver *P* = n.s.; liver harbouring carcinoma vs control liver *P* = n.s.).

As in the case of spontaneous hepatic tumours, the nuclear and cytoplasmic basal MAPK activities in the implanted liver

© 2000 Cancer Research Campaign

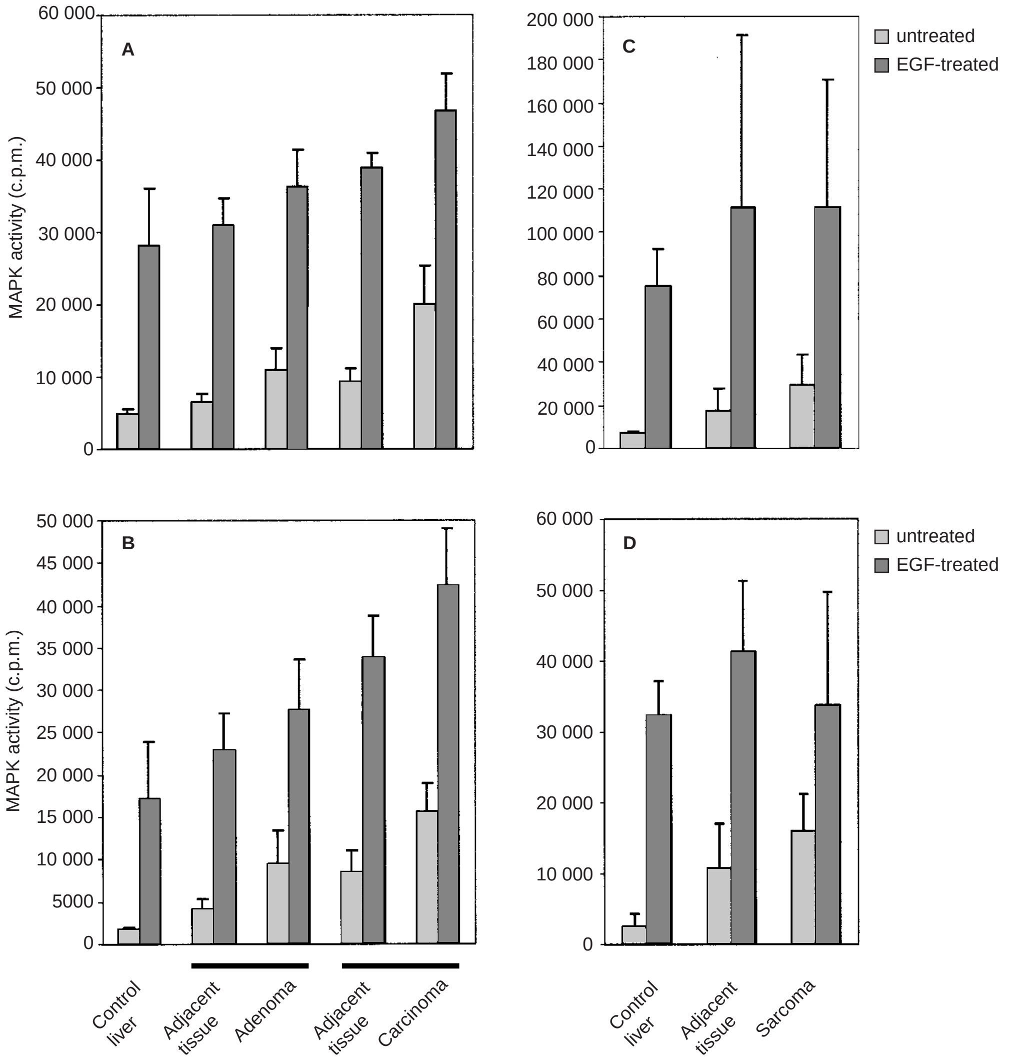

**Figure 1** MAPK activities in hepatic spontaneous tumours, livers harbouring tumours and livers from control CBA-T6/W mice (**A, B**) and in hepatic-implanted sarcomas, livers harbouring tumours and livers from control BALB/c mice (**C, D**) after intraperitoneal injection of either saline or EGF. CBT-T6/W mice with spontaneous hepatocellular neoplasm and BALB/c mice with liver implanted L1 sarcoma tumours as well as control 24-month-old CBA-T6/W or sham-operated BALB/c mice were used. Ten minutes after intraperitoneal injection of either saline or EGF (10 μg $g^{-1}$ of body weight), animals were anaesthetized, livers were harvested, classified into tumour or normal hepatic tissue and were frozen in liquid nitrogen until use. Cytosolic (**A**, **C**) and nuclear (**B**, **D**) extracts were prepared and immunocomplex kinase assays were performed (see Methods). Results are shown for MAPK activities in livers from normal animals (control), in the pair of adenomas (adenoma) and the harbouring livers (adjacent tissue), in the pair of carcinomas (carcinoma) and the harbouring livers (adjacent tissue), and in the pair of sarcomas (sarcoma) and the harbouring livers (adjacent tissue). Results represent means ± s.d. of six pairs of control CBA-T6/W and six pairs of control BALB/c animals, seven pairs of animals with hepatocellular adenomas, four pairs of animals with hepatocellular carcinomas and ten pairs of animals with implanted sarcomas.

sarcomas in BALB/c mice were higher than the MAPK activities in the normal tissue of the liver harbouring these tumour (Figure 1) (CE: sarcoma vs adjacent liver $P < 0.05$), (NE: sarcoma vs adjacent liver $P < 0.05$). Also, the MAPK activities in the normal tissues from livers harbouring these sarcomas were several-fold higher than the activities of MAPK in the livers of sham-operated BALB/c mice (CE: liver harbouring sarcoma vs control liver $P < 0.01$), (NE: liver harbouring sarcoma vs control liver $P < 0.01$). After treatment with EGF, cytoplasmic and nuclear MAPK activities increased several-fold, and in all the tissues MAPK activities reached the same high levels (CE: sarcoma vs adjacent liver $P$ = n.s.), (NE: sarcoma vs adjacent liver $P$ = n.s.), (CE: liver harbouring sarcoma vs control liver $P$ = n.s.), (NE: liver harbouring sarcoma vs control liver $P$ = n.s.).

### p70S6k activities in hepatic tumours, livers harbouring tumours and livers from control mice after intraperitoneal injection of either saline or EGF

Immunocomplex kinase assays were carried out to assess the activities of p70S6k (Figure 2) in the same tissues that were used to measure MAPK activities (Figure 1). As in the case of MAPK, the basal activities of p70S6k in spontaneous hepatic tumours were higher than those measured in the adjacent normal liver

*© 2000 Cancer Research Campaign*

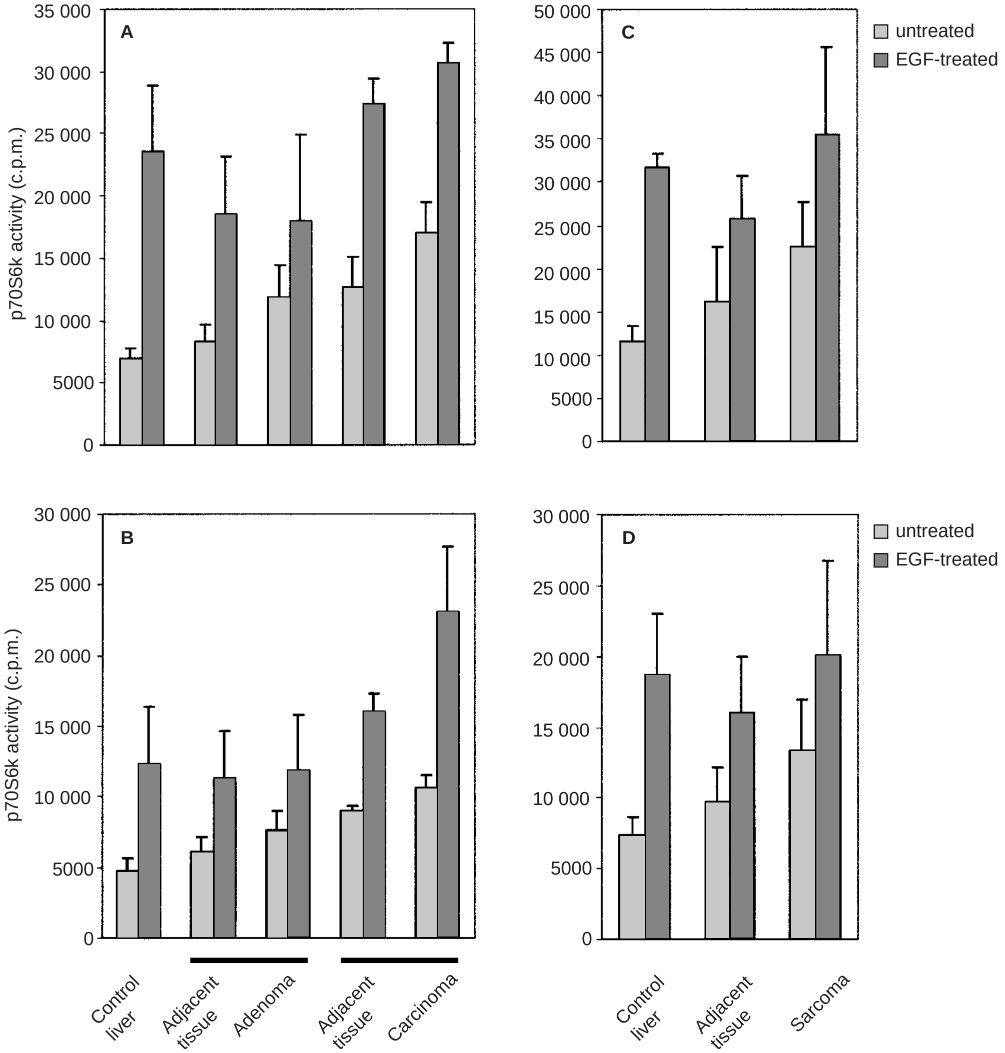

**Figure 2** p70S6k activities in hepatic spontaneous tumours, livers harbouring tumours and livers from control CBA-T6/W mice (**A, B**) and in hepatic-implanted sarcomas, livers harbouring tumours and livers from control BALB/c mice (**C, D**) after intraperitoneal injection of either saline or EGF. Cytosolic (**A**, **C**) and nuclear (**B**, **D**) extracts were prepared and immunocomplex kinase assays were used to assess p70S6k activities as described in Fig. 1. Results represent means ± s.d.

tissue or livers from control animals (CE: adenoma vs adjacent liver $P < 0.05$; carcinoma vs adjacent liver $P < 0.05$), (NE: adenoma vs adjacent liver $P < 0.05$; carcinoma vs adjacent liver $P < 0.05$), (CE: adenoma vs control liver $P < 0.05$; carcinoma vs control liver $P < 0.01$), (NE: adenoma vs control liver $P < 0.05$; carcinoma vs control liver $P < 0.01$). Also, the p70S6k activities in the livers harbouring the neoplasms were higher that the activity of this enzyme measured in the livers of control animals without tumour (CE: liver harbouring adenoma vs control liver $P < 0.05$; liver harbouring carcinoma vs control liver $P < 0.01$), (NE: liver harbouring adenoma vs control liver $P < 0.05$; liver harbouring carcinoma vs control liver $P < 0.01$). EGF treatment stimulated p70S6k activities in tumours and normal tissues to the same level but the degree of the fold-increase was not as large as that seen with the EGF-inducible MAPK activity (compare Figures 1 and 2) (CE: adenoma vs adjacent liver $P$ = n.s.; carcinoma vs adjacent liver $P$ = n.s.), (NE: adenoma vs adjacent liver $P$ = n.s.; carcinoma vs adjacent liver $P$ = n.s.), (CE: adenoma vs control liver $P$ = n.s.; carcinoma vs control liver $P$ = n.s.), (NE: adenoma vs control liver $P$ = n.s.; carcinoma vs control liver $P$ = n.s.), (CE: liver harbouring adenoma vs control liver $P$ = n.s.; liver harbouring carcinoma vs control liver $P$ = n.s.), (NE: liver harbouring adenoma vs control liver $P$ = n.s.; liver harbouring carcinoma vs control liver $P$ = n.s.).

As in the case of spontaneous hepatic tumours, the nuclear and cytoplasmic p70S6k activities in the implanted liver sarcomas in saline-treated BALB/c mice were higher than the p70S6k activities in the normal tissue of the livers with implanted tumours (Figure 2) (CE: sarcoma vs adjacent liver $P < 0.05$), (NE: sarcoma vs adjacent liver $P < 0.05$). Also, the p70S6k activities in the normal tissues from livers harbouring the sarcomas were significantly higher than the activities of this enzyme in the livers of sham-operated BALB/c mice (CE: liver harbouring sarcoma vs control liver $P < 0.05$), (NE: liver harbouring sarcoma vs control liver $P < 0.05$). After treatment with EGF, cytoplasmic and nuclear

© 2000 Cancer Research Campaign

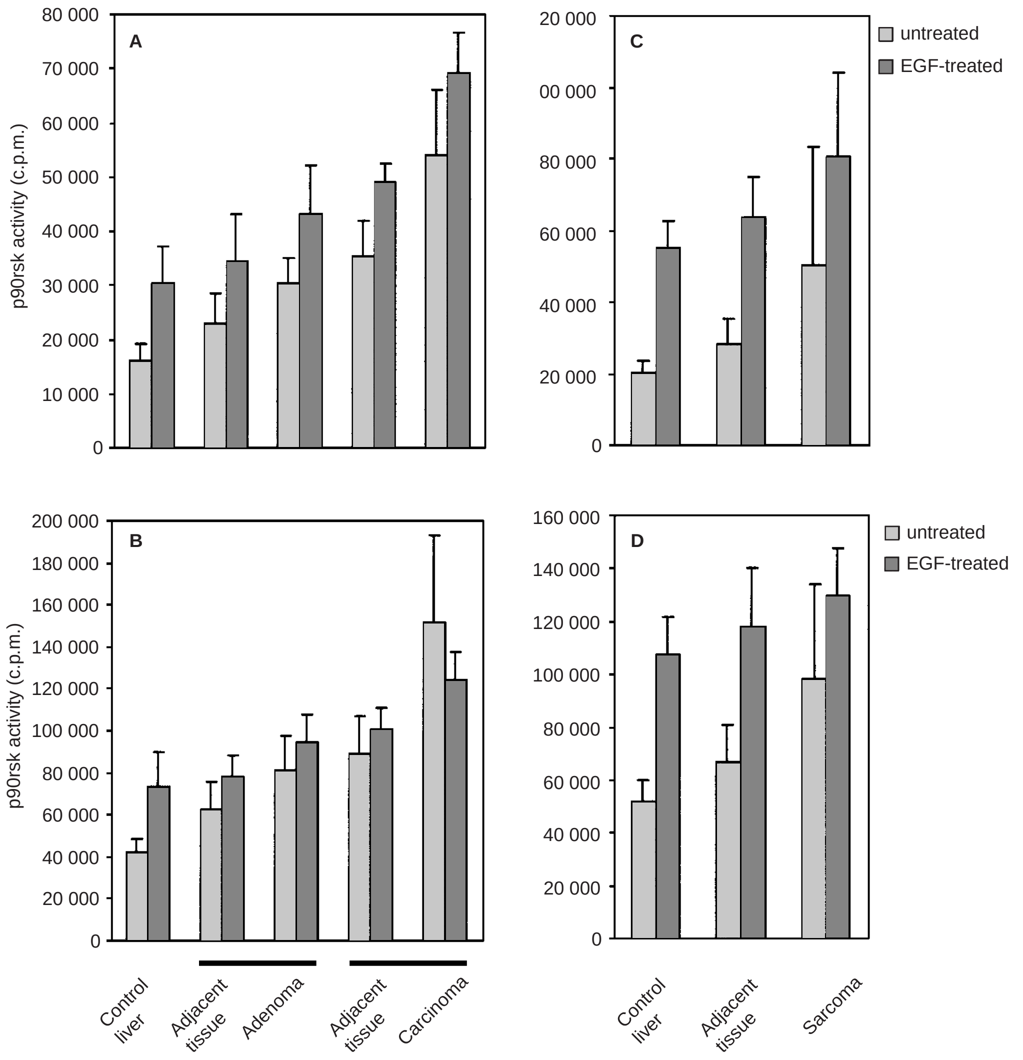

**Figure 3** p90rsk activities in hepatic spontaneous tumours, livers harbouring tumours and livers from control CBA-T6/W mice (**A, B**) and in hepatic-implanted sarcomas, livers harbouring tumours and livers from control BALB/c mice (**C, D**) after intraperitoneal injection of either saline or EGF. Cytosolic (**A, C**) and nuclear (**B, D**) extracts were prepared and immunocomplex kinase assays were used to assess p90rsk activities as described in Figure 1. Results represent means ± s.d p70S6k activities increased several-fold in the normal tissues and in the sarcomas and achieved approximately the same absolute level (CE: sarcoma vs adjacent liver $P$ = n.s.), (NE: sarcoma vs adjacent liver $P$ = n.s.), (CE: liver harbouring sarcoma vs control liver $P$ = n.s.), (NE: liver harbouring sarcoma vs control liver $P$ = n.s.).

### p90rsk activities in hepatic tumours, livers harbouring tumours and livers from control mice after intraperitoneal injection of either saline or EGF

Immunocomplex kinase assays were carried out to assess the activities of cytoplasmic and nuclear p90rsk (Figure 3) in the same tissues used to measure MAPK (Figure 1) and p70S6k activities (Figure 2).

Similar to the other two enzymes, p90rsk activities were higher in the spontaneous and implanted tumours than in the adjacent normal liver tissues or normal control livers (CE: adenoma vs adjacent liver $P < 0.05$; carcinoma vs adjacent liver $P < 0.05$; sarcoma vs adjacent liver $P < 0.05$), (NE: adenoma vs adjacent liver $P < 0.05$; carcinoma vs adjacent liver $P < 0.05$; sarcoma vs adjacent liver $P < 0.05$), (CE: adenoma vs control liver $P < 0.01$; carcinoma vs control liver $P < 0.01$; sarcoma vs control liver $P < 0.05$), (NE: adenoma vs control liver $P < 0.05$; carcinoma vs control liver $P < 0.01$; sarcoma vs control liver $P < 0.05$). Similar to the other two kinases, p90rsk activities in the adjacent liver tissue were higher than in the livers in the normal animals, (CE: liver harbouring adenoma vs control liver $P < 0.05$; liver harbouring carcinoma vs control liver $P < 0.05$; liver harbouring sarcoma vs control liver $P < 0.05$), (NE: liver harbouring adenoma vs control liver $P < 0.01$; liver harbouring carcinoma vs control liver$P < 0.01$; liver harbouring sarcoma vs control liver $P < 0.01$). Also, as with MAPK and p70S6k, the EGF-induced p90rsk activities reached approximately the same absolute level in the liver of control animals, livers harbouring the tumours and the tumours themselves. (CE: adenoma vs adjacent $P$ = n.s.; carci-

*© 2000 Cancer Research Campaign*

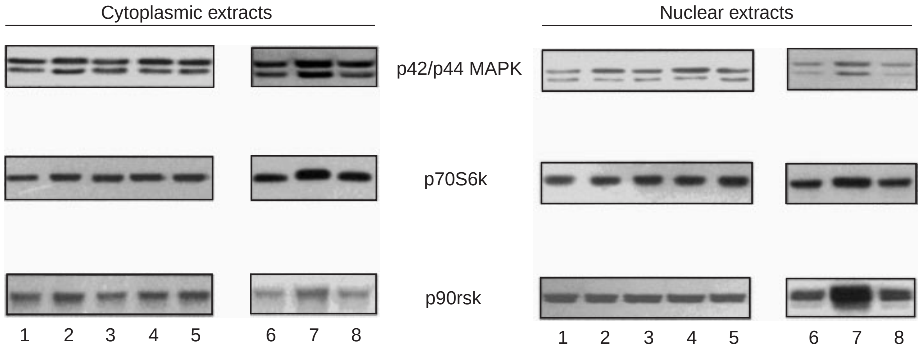

**Figure 4** Immunoblotting of cytosolic and nuclear extracts from hepatic spontaneous tumours, implanted sarcomas, tumour harbouring livers and livers from normal control animals (CBA-T6/W or sham-operated BALB/c mice). Equal amounts of protein (100 µg) were separated by SDS-PAGE, proteins were transferred to PVDF membrane and probed with specific antibodies to MAPK, p70S6k and p90rsk. Results are from single experiments representative of at least three experiments giving similar results. 1. normal liver from CBA-T6/W; 2. adenoma; 3. liver harbouring adenome; 4. cacinoma; 5. liver harbouring carcinoma; 6. normal liver from BALB/C; 7. sarcoma; 8. liver harbouring sarcoma

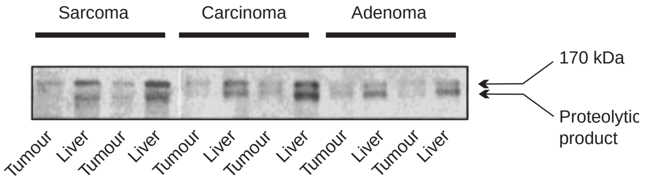

**Figure 5** Identification of EGFR purified from hepatic spontaneous tumours, implanted sarcomas and liver harbouring tumours. Equal amounts of cytoplasmic protein (100 µg) were separated by SDS-PAGE, proteins were transferred to PVDF membrane and probed with specific antibodies to EGFR. Results are representative of at least three giving similar results.

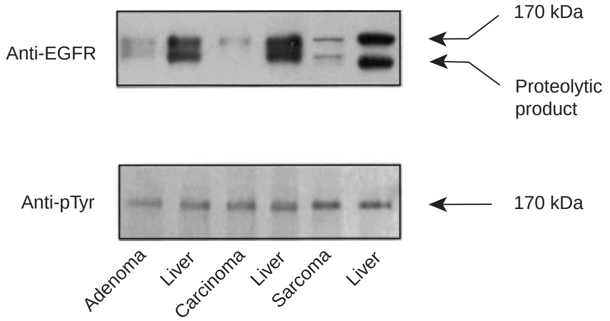

**Figure 6** Tyrosine phosphorylation of EGFR purified from hepatic spontaneous tumours, implanted sarcomas and tumour harbouring livers. Equal amounts of cytoplasmic protein (1 mg) were immunoprecipitated with anti-EGFR antibodies. Proteins, eluted from protein A/G by boiling in loading buffer, were separated by SDS-PAGE, transferred to PVDF membrane, and probed with either specific antibodies to EGFR (upper panel) or anti-phosphotyrosine (anti-pTyr) (lower panel). Results are representative of three experiments giving similar results.

noma vs adjacent $P < 0.05$; sarcoma vs adjacent liver $P$ = n.s.), (NE: adenoma vs adjacent $P$ = n.s.; carcinoma vs adjacent $P < 0.05$; sarcoma vs adjacent liver $P$ = n.s.), (CE: adenoma vs control liver $P$ = n.s.; carcinoma vs control liver $P < 0.05$; sarcoma vs control liver $P$ = n.s.), (NE: adenoma vs control liver $P$ = n.s.; carcinoma vs control liver $P < 0.05$; sarcoma vs control liver $P$ = n.s.), (CE: liver harbouring adenoma vs control liver $P$ = n.s.; liver harbouring carcinoma vs control liver $P$ = n.s.; liver harbouring sarcoma $P$ = n.s.), (NE: liver harbouring adenoma vs control liver $P$ = n.s.; liver harbouring carcinoma vs control liver $P$ = n.s.; liver harbouring sarcoma vs control liver $P$ = n.s.). However, compared to the other two kinases, the profiles of p90rsk activities were different in two important ways. First, in all tissues examined the basal and EGF-inducible nuclear p90rsk activities were higher than those measured in the cytoplasm. Second, in the CBA-T6/W animals (Figure 3) the EGF-response of p90rsk was either small or not demonstrable at all, particularly in the nuclear fractions.

In summary, these results show that the basal MAPK, p70S6k and p90rsk activities are elevated in several hepatic tumours but in most instances the basal activities were significantly below the MAPK, p70S6k and p90rsk activities that were induced by systemic administration of EGF. The basal, but not the EGF-induced, activities of these enzymes appear to correlate with histology of the tumour with the higher levels measured in the carcinomas and sarcomas compared to adenomas. Unexpectedly, the basal, but not the EGF-induced, MAPK, p70S6k and p90rsk activities in normal tissue of livers harbouring these tumours were greater than the activities measured in livers from control animals.

### MAPK, p70S6k and p90rsk protein levels in hepatic tumours and livers harbouring tumours and livers from control mice

Immunoblot analyses of the same total amount of cytoplasmic and nuclear protein extracts were carried out to determine whether the increased kinase activities reflected changes in the kinase protein levels. As shown in Figure 4, detectable levels of cytoplasmic and nuclear MAPK, p70S6k and p90rsk were found, which did not differ significantly between extracts from spontaneous tumours, livers harbouring tumours and livers from control animals. Thus, kinase activation of these enzymes in adenomas and adenocarcinomas and the adjacent liver tissue does not seem to reflect net increase in kinase protein levels but rather may reflect protein modification, most likely phosphorylation. Since the total amount of proteins extracted from the cytoplasm is several-fold higher than the total amount of protein extracted from the hepatic nuclei, the total levels of these kinases is several-fold higher in the cytoplasm than in the nucleus. In contrast, Western blots revealed

*© 2000 Cancer Research Campaign*

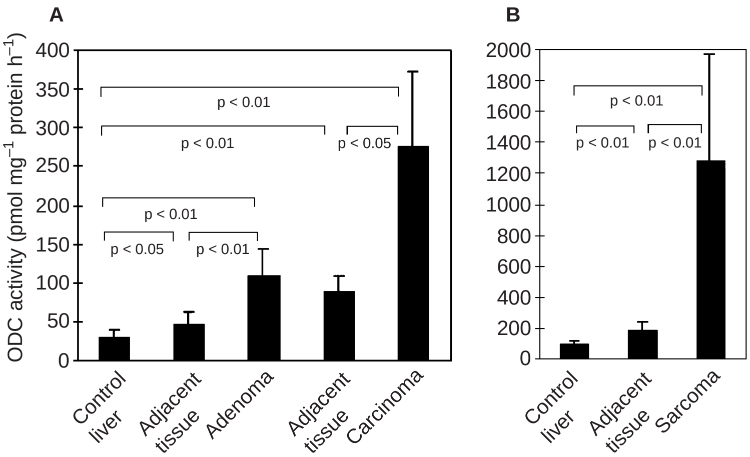

**Figure 7** ODC activities in hepatic spontaneous tumours, livers harbouring tumours and livers from control CBA-T6/W mice (**A**) and in hepatic-implanted sarcomas, livers harbouring tumours and livers from control BALB/c mice (**B**). ODC activities were measured using L-[$^{14}$C]-ornithine hydrochloride as a substrate in hepatocellular adenomas ($n$ = 7), hepatocellular carcinomas ($n$ = 4) and sarcomas implanted into liver ($n$ = 10). For comparison, ODC activities were also measured in normal livers harbouring tumours and in livers from control animals. Results represent means ± s.d.

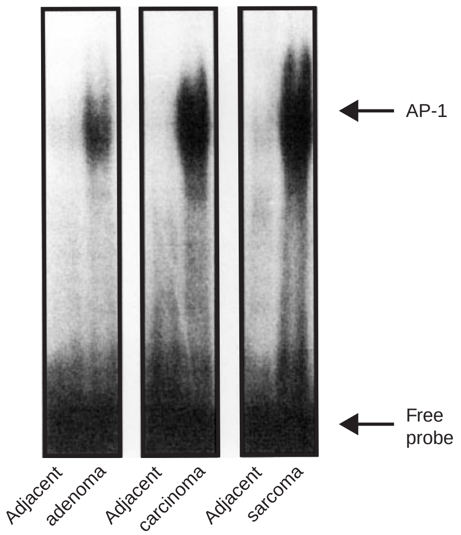

**Figure 8** Activation of AP-1 DNA-binding activity in liver tumours. AP-1 DNA-binding activity was assayed using electrophoretic mobility shift assay in nuclear extracts derived from either hepatocellular adenoma, hepatocellular carcinoma and sarcoma implanted into livers or the liver around tumours.

increased protein amounts of MAP, p70S6 and p90rs kinases in cytoplasmic and nuclear extracts from implantable sarcomas comparing to the extracts from both livers harbouring sarcomas and livers from normal control animals.

### EGF receptor levels in hepatic tumours and livers harbouring tumours

EGFR protein levels in cell extracts from spontaneous hepatocellular neoplasms, implanted L1 sarcoma tumours, and normal livers adjacent to tumours were assessed by Western blotting analysis using anti-EGFR antibodies. The results showed (Figure 5) that in each pair of tumour and tumour-harbouring liver, the liver tissue contained higher level of 170 kDa EGFR than that detected in the corresponding neoplasms. These results, which are in agreement with studies previously reported by Yang and co-workers (Yang et al, 1994) in rats with transplantable tumours, suggest that the EGFRs in liver neoplasms are down-regulated. An additional band of lower molecular weight (approximately 150 kDa) was also found in both tumours and adjacent livers; this band likely represents a proteolytic product of 170 kDa receptor (Yang et al, 1994). Interestingly, proteolytic products of EGFR in both sarcomas and sarcoma-harbouring liver have faster electrophoretic mobility than those found in spontaneous tumours and the adjacent livers. In contrast to differences in EGFR protein levels, the anti-phosphotyrosine blots revealed that the intensity of a 170 kDa band of EGFR was similar in immunoprecipitated extracts from hepatic tumours and the adjacent livers (Figure 6). The putative proteolytic product of the EGFR was not tyrosine-phosphorylated. While these results demonstrate that the total number of phosphotyrosines present in EGFRs is similar in both tissues, the stoichiometry of tyrosine phosphorylation in normal tissues might be lower.

### Constitutive activity of ODC is increased in the liver tumours and in the normal liver tissue that harbours these tumours

We next assessed the ODC activity in these tumours since this enzyme is thought to play a role in the mitogenic responses. In old CBA-T6 and young BALB/c mice ODC activity in livers from normal animals averaged 29 ± 11 and 95 ± 28 pmol h$^{-1}$ mg$^{-1}$ protein respectively (Figure 7). ODC activity in spontaneous neoplasms and implanted sarcomas were greater than the activity of ODC in normal tissue of the livers bearing the tumours. Compared to the normal tissue, the ODC activity in the tumours was two- to eightfold higher, with the highest differences observed between normal tissue and the implanted sarcomas. As in the case of kinases, ODC activities in the normal tissue harbouring the tumour was significantly higher than the activity measured in the liver extracts from control mice (Figure 7).

 © 2000 Cancer Research Campaign

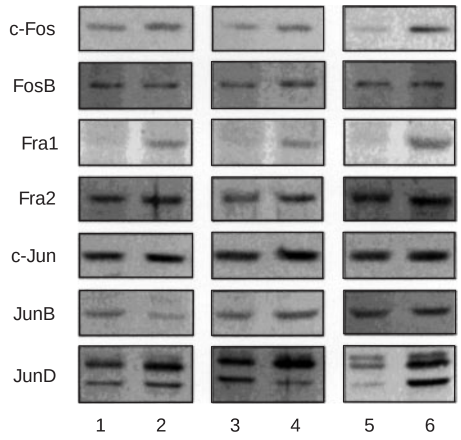

**Figure 9** Western blot analyses of AP-1 nuclear proteins in hepatic spontaneous tumours, implanted sarcomas and tumour harbouring livers. Nuclear extracts (100 μg) were fractionated by SDS-PAGE and transferred to PVDF membranes. Immunoblots were performed with antibodies specific for c-Fos, FosB, Fra-1, Fra-2, c-Jun, JunB and JunD. Results are from single experiments representative of at least three experiments giving similar results 1. adenoma; 2. liver harbouring adenoma; 3. carcinoma; 4. liver harbouring carcinoma; 5. sarcoma; 6. liver harbouring sarcoma.

### AP-1 DNA-binding activity is increased in hepatic tumours but not in the livers that harbour these tumours

AP-1 is a transcription factor that is involved in mitogenic processes, including oncogenesis. AP-1 is activated by a number of growth factors, including EGF. We have recently shown that systemic administration of EGF activates AP-1 DNA-binding activity in nuclei of mouse livers (Ostrowski et al, 2000). The growth factor-triggered AP-1 activation is mediated by the MAP kinase cascades. As is shown in Figure 8, AP-1 DNA-binding activity is increased in nuclear extracts from the neoplasms, but unlike the enzyme activities (Figures 1–3 and 7) the increased AP-1 DNA-binding activity does not correlate with the tumours' histology. Also, unlike the enzymes that we studied, no activation of AP-1 DNA-binding activity was found in the nuclear extracts derived from the normal tissue of the livers that had the tumours.

The nuclear levels of the individual members of the Fos (c-Fos, FosB, Fra-1 and Fra-2) and Jun (c-Jun, JunB and JunD) family were assessed by immunoblot analyses of nuclear extracts prepared from hepatic neoplasms and tumour harbouring livers. As shown in Figure 9, detectable levels of Fos and Jun nuclear proteins were found in spontaneous and implanted hepatic tumours as well as in adjacent normal livers. The nuclear protein concentration of c-Fos, FosB, Fra2, c-Jun and JunB were very similar in the tumours and the tumour harbouring livers, whereas some increased accumulation of Fral and JunD was found in normal livers as compared to liver tumours.

### DISCUSSION

This study shows that the activities of MAPK, p70S6k and p90rsk are increased in hepatocellular neoplasms. We also demonstrated elevated activity of ODC in these neoplasms. The enhanced MAPK activity in hepatic tumours is consistent with similar observations made in human and rat colorectal cancers (Licato et al, 1997; Ostrowski et al, 1998), renal cell carcinoma (Oka et al, 1995), prostate cancer (Magi-Galluzzi et al, 1997), and a subset of acute, but not chronic myelogenous leukaemias (Towatari et al, 1997). However, increased activities of p70S6k and p90rsk have not been previously reported. To our knowledge this is also the first demonstration that kinases and ODC activities are increased in tissues surrounding tumours.

MAPK, p90rsk and p70S6k are normally activated by ligand binding to the cell surface receptors (Lewis et al, 1998). In oncogenic processes these kinases can also be activated by mutated intracellular mediators that are constitutively driving these pathways. A well described example of such an oncogenic process is mutated *RAS* (Santos et al, 1984). Since the signal transduction pathways that activate MAPK and p70S6k are fundamentally different, the observation that these kinases are concurrently activated suggests that the triggers for their activation in these tumours are located at the earliest tiers of the signal transduction pathways. In fact, it is conceivable that a trigger point is located at the level of growth factor receptor itself where its activation might be ligand-mediated. For example, the EGFR could be driven by EGF. Such a possibility is consistent with our observation that there is a concurrent basal activation of MAPK, p90rsk and p70S6k in hepatic tumours that is reminiscent of the profile of kinase activation by EGF (Ostrowski et al, 2000) (Figures 1–3).

If the above postulate is correct, what is the source of EGF (or another mitogen)? Malignant cells are able to synthesize several cytokines and growth factors sometimes acting in an autocrine stimulatory fashion, which – following extracellular release – might function primarily as para/endocrine stimuli. It is therefore conceivable that, in part, the activation of the MAPK, p90rsk and p70S6k is mediated by EGF and/or other growth factors that are secreted by the tumour. Two observations made in this study are consistent with such a possibility:

1. MAPK, p90rsk and p70S6k activities were consistently higher in the liver surrounding the tumours compared to livers from control animals. Although there are other explanations, the elevated MAPK, p90rsk and p70S6k activities may reflect increased production of EGF, and/or other growth factors, by hepatic tumours that reach other parts of the involved liver.
2. EGFR level was significantly lower in tumours compared to liver harbouring tumour suggesting EGF-mediated receptor down-regulation. Systemic administration of EGF stimulated MAPK, p90rsk and p70S6k not only in normal livers but also in the tumours. Regardless of histology, the EGF-induced activities achieved approximately the same levels in all the tissues examined. This means that the tumours are fully responsive to EGF in a way similar to normal tissue.

If paracrine secretion by the tumours is responsible for the elevation of kinase activities, then it is conceivable that EGF and/or other mitogens could be contributing to the generation and maintenance of these malignant processes. Even if paracrine secretion of EGF does play a role, EGF is surely only one of many factors contributing to these malignancies. The observation that the liver surrounding the tumour was histologically normal yet it had elevated kinase activities support the notion that activation of these enzyme is not in a simple relation to hepatic malignancy. It is conceivable that AP-1 along with other transcription factors are key components responsible for the enhanced mitogenic state of the tumours. It is conceivable that a given threshold of kinase

© 2000 Cancer Research Campaign activities is required to trigger AP-1, which (in conjunction with many other factors) induces the oncogenic process.

In summary, our study demonstrates a significant basal activation of MAP, p90rs and p70S6 kinases and high constitutive AP-1 DNA binding activity in mouse spontaneous hepatic adenomas and carcinomas. The activities of kinases, but not that of AP-1, were also increased in surrounding liver tissue. Unlike the different basal activities, the EGF-triggered kinase responses in normal liver and liver tumours were indistinguishable. These observations open up a new avenue to explore the intracellular mechanisms responsible for the generation and maintenance of hepatic tumours.

### ACKNOWLEDGEMENTS

This work was supported by grant from the Polish Committee for Scientific Research (KBN 4 PO5A 114 10) (to JO).

### REFERENCES

Banerjee P, Ahmad MF, Grove JR, Kozlosky C, Price DJ and Avruch J (1990) Molecular structure of a major insulin/mitogen-activated 70-kDa S6 protein kinase. *Proc Natl Acad Sci USA* **87**: 8550–8554

Boulikas T (1995) Phosphorylation of transcription factors and control of the cell cycle. *Crit Rev Eukaryotic Gene Exp* **5**: 1–77

Carpenter G and Cohen S (1981) Epidermal growth factor. *J Biol Chem* **265**: 7709–7712

Decker S (1980) Phosphorylation of ribosomal protein S6 in avian sarcoma virus-transformed chicken embryo fibroblasts. *Proc Natl Acad Sci USA* **78**: 4112–4115

Dignam JO, Lebovitz RM and Roeder R (1998) Accurate transcription initiation by RNA polymerase II in a soluble extract from isolated mammalian nuclei. *Nucleic Acids Res* 1983; **11**: 1475–1489

Downward J (1998) Lipid-regulated kinases: some common themes at last. *Science* **279**: 673–674

Fausto N, Laird AD and Webber EM (1995) Role of growth factors and cytokines in hepatic regeneration. *FASEB J* **9**: 1527–1536

Frith CH, Ward JM and Turusov VS (1994) Tumours of the liver. In: *Pathology of Tumours in Laboratory Animals*, Turusov V and Mohr U (eds), pp. 223–248. IARC Scientific Publications No. 111 IARC: Lyon

Huber BE, Heilman CA and Thorgeirsson SS (1989) Poly(A+) RNA levels of growth-, differentiation- and transformation-associated genes in the progressive development of hepatocellular carcinoma in the rat. *Hepatology* **9**: 756–762

Janik P, Bertram JS and Szaniawska B (1981) Modulation of lung tumor colony formation by a subcutaneously growing tumor. *J Natl Cancer Inst* **66**: 1155–1158

Kozma SC, Ferrari S, Bassand P, Siegmann M, Totty N and Tho G (1990) Cloning of the mitogen-activated s6 kinase from rat liver reveals an enzyme of the second messenger subfamily. *Proc Natl Acad Sci USA* **87**: 7365–7369

Lee W, Haslinger A, Karin M and Tjian R (1987) Activation of transcription by two factors that bind promoter and enhancer sequences of the human metallothionein gene and SV 40. *Nature* **325**: 368–372

Levitzki A (1996) Targeting signal transduction for disease therapy. *Curr Opin Cell Biol* **8**: 239–242

Lewis TS, Shapiro PS and Ahn NG (1998) Signal transduction through MAP kinase cascades. *Adv Cancer Res* **74**: 49–139

Licato LL, Keku TO, Wurzelmann JI, Murray SC, Woosley JT, Sandler RS et al (1997) In vivo activation of mitogen-activated protein kinases in rat intestinal neoplasia. *Gastroenterology* **113**: 1589–1598

McGowan JA, Strain AJ and Bucher NLR (1981) DNA synthesis in primary cultures of adult rat hepatocytes in a defined medium: effects of epidermal growth factor, insulin, glucagon, and cyclic-AMP. *J Cell Physiol* **108**: 353–363

Magi-Galluzzi C, Mishra R, Fiorentino M, Montironi R, Yoa H, Capodieci P et al (1997) Mitogen-activated protein kinases phosphatase 1 is overexpressed in prostate cancers and is inversely related to apoptosis. *Lab Invest* **76**: 37–51

Marshall CJ (1994) MAP kinase kinase kinase, MAP kinase kinase and MAP kinase. *Curr Topics Genet Dev* **4**: 82–89

Moller D, Xia C-H, Tang W, Zhu AX and Jakubowski M (1994) Human rsk isoforms: cloning and characterization of tissue-specific expression. *Am J Physiol* **266**: C351–C359

Mullhaupt B, Feren A, Fodor E and Jones A (1994) Liver expression of epidermal growth factor RNA. Rapid increases in immediate-early phase of liver regeneration. *J Biol Chem* **269**: 19667–19670

Nadori F, Lardeux B, Rahmani M, Bringuier A, Durand-Schneider A-M and Bernuau D (1997) Presence of distinct AP-1 dimers in normal and transformed rat hepatocytes under basal conditions and after epidermal growth factor stimulation. *Hepatology* **26**: 1477–1483

Oka H, Chatani Y, Hoshino R, Ogawa O, Kakehi Y, Terachi T et al (1995) Constitutive activation of mitogen-activated protein (MAP) kinases in human renal cell carcinoma. *Cancer Res* **55**: 4182–4187

Ostrowski J, Sims JE, Sibley CH, Valentine MA, Dower SK, Meier KE et al (1991) A serine/threonine kinase activity is closely associated with a 65-kDa phosphoprotein specifically recognized by the Kβ enhancer element. *J Biol Chem* **266**: 12722–12733

Ostrowski J, Wojciechowski K, Konturek SJ and Butruk E (1993) Inhibitory effect of EGF on secretory response of parietal cells is associated with an induction of ODC. *Am J Physiol* **264**: (Cell Physiol.): C1428–C1433

Ostrowski J, Trzeciak L, Kolodziejski J and Bomsztyk K (1998) Increased constitutive activity of MAP kinase and renaturable 85 kDa kinase in human colorectal cancer. *Br J Cancer* **78**: 1301–1306

Ostrowski J, Woszczynski M, Kowalczyk P, Trzeciak L, Henning E and Bomsztyk K (2000) Treatment of mice with EGF and orthovanadate activates cytoplasmic and nuclear MAPK, p70S6k, and p90rsk in the liver. *J Hepatol* (in press)

Pegg AE (1986) Recent advances in the biochemistry of polyamines in eukaryotes. *Biochem J* **234**: 249–262

Pullen N and Thomas G (1997) The modular phosphorylation and activation of p70S6k. Minireview. *FEBS Lett* **410**: 78–82

Santos E, Martin-Zanca D, Reddy EP, Pierotti MA, Della Porta G and Barbacid M (1984) Malignant activation of a K-ras oncogene in lung carcinoma but not in normal tissue of the same patient. *Science* **223**: 661–664

Sturgill TW and Wu J (1991) Recent progress in characterization of protein kinase cascades for phosphorylation of ribosomal protein S6. *Biochim Biophys Acta* **1092**: 350–357

Tamori A, Nishiguchi S, Kuroki T, Seki S, Kobayashi K, Kinoshita H et al (1994) Relationship of ornithine decarboxylase activity and histological findings in human hepatocellular carcinoma. *Hepatology* **20**: 1179–1186

Thomas G, Siegmann M and Gordon J (1979) Multiple phosphorylation of ribosomal protein S6 during transition of quiescent 3T3 cells into early G1, and cellular compartmentalization of the phosphate donor. *Proc Natl Acad Sci USA* **76**: 3952–3956

Towatari M, Lida H, Tanimoto M, Iwata H, Hamaguchi M and Saito H (1997) Constitutive activation of mitogen-activated protein kinase pathway in acute leukemia cells. *Leukemia* **11**: 479–484

Westwick JK, Fleckenstein J, Yin M, Yang SQ, Bradham CA, Brenner DA et al (1996) Differential regulation of hepatocyte DNA synthesis by cAMP in vitro and in vivo. *Am J Physiol* **271**: G780–G790

Williams-Ashman HG, Coppoc LL and Weber G (1972) Imbalance ornithine metabolism in hepatomas of different growth rates expressed in formation of putrescine, spermidine, and spermine. *Cancer Res* **32**: 1924–1932

Yang E-B, Mack P and Cheng L-Y (1995) Studies of epidermal growth factor (EGF) receptor in plasma membrane from rat liver and hepatoma tissues. *Biochem Mol Biol Intl* **33**: 221–228

 *© 2000 Cancer Research Campaign*
